# A Radiographic Study on the Associations of Age and Prevalence of Vertebral Fractures with Abdominal Aortic Calcification in Japanese Postmenopausal Women and Men

**DOI:** 10.4061/2010/748380

**Published:** 2009-12-20

**Authors:** Jun Iwamoto, Hideo Matsumoto, Tsuyoshi Takeda, Yoshihiro Sato, Mitsuyoshi Uzawa

**Affiliations:** ^1^Institute for Integrated Sports Medicine, Keio University School of Medicine, 35 Shinanomachi, Shinjuku-ku, Tokyo 160-8582, Japan; ^2^Department of Neurology, Mitate Hospital, Fukuoka, Tagawa 826-0041, Japan; ^3^Department of Orthopaedic Surgery, Keiyu Orthopaedic Hospital, Gunma 374-001, Japan

## Abstract

The purpose of the present study was to determine the associations of age and history of non- and low-traumatic fractures with the severity of abdominal aortic calcification in Japanese postmenopausal women and men. Four hundred and one Japanese persons (24 men and 377 postmenopausal women, mean age: 73.8 years) for whom thoracic and lumbar spine radiographs had been obtained to evaluate their posture prior to patient participation in a fall-prevention exercise program were enrolled. The associations of sex, age, history of hip fracture, prevalence of vertebral fracture, and spondylosis grade (the Nathan degree) with the severity of abdominal aortic calcification (length of calcification, as evaluated according to the number of vertebral bodies) were analyzed. Nine subjects (2.2%) had a history of hip fracture, and 221 (55.1%) had at least one prevalent vertebral fracture. Two hundred and sixty-seven subjects (66.6%) had first-degree spondylosis. Age and the number of prevalent vertebral fractures, but not sex, history of hip fracture, or spondylosis grade, were significantly associated with the severity of abdominal aortic calcification. The present study confirmed that age and the number of vertebral fractures were associated with the severity of abdominal aortic calcification in Japanese postmenopausal women and men.

## 1. Introduction

Both vascular calcification and osteoporosis increase with age and are commonly observed in the elderly. Abdominal aortic calcification is prominently displayed on routine lateral lumbar spine radiographs as the dense calcium mineral deposits in the aorta that lie adjacent to the osteoporotic vertebrae. Aortic calcification detected on radiographs is considered to be indicative of late-stage atherosclerosis [1, 2]. It is intimately associated with atherosclerotic plaque development [3] and is an independent predictor of subsequent vascular morbidity and mortality [1, 2].

Although the associations of age and bone mineral density (BMD) with aortic calcification have been well examined [4–10], whether osteoporosis and aortic calcification are related to each other or are independent, age-related processes remain uncertain. The absolute level of BMD is predictive of fracture risk, and a gradient of increasing fracture risk is associated with declining levels of BMD [11]. However, because the BMD T scores vary among skeletal sites, the history of non- or low-traumatic fractures, such as hip and vertebral fractures, may be a useful measure for assessing the severity of osteoporosis.

A few studies have shown a relation between aortic calcification and hip fractures in healthy postmenopausal European women, although the results for vertebral fractures are controversial [4, 12]. However, the incidence of hip and vertebral fractures may differ between Japanese and Caucasians. Namely, the incidence of hip fractures is lower but that of spine fractures is greater among Japanese than among Caucasians [13, 14]. The association between aortic calcification and osteoporosis based on non- or low-traumatic vertebral fractures remains to be established in Japanese subjects. The purpose of the present study was to determine the associations of age, history of hip fracture, and the prevalence of vertebral fractures with the severity of abdominal aortic calcification in postmenopausal women and men.

## 2. Subjects and Methods

### 2.1. Subjects

Four hundred and one Japanese persons (24 men and 377 postmenopausal women) who participated in a fall-prevention exercise program at Keiyu Orthopaedic Hospital (Gunma, Japan) during the 3 years between September 2005 and August 2008 were assessed. Subjects with diseases that could significantly affect bone metabolism, including primary hyperparathyroidism, thyroid dysfunction, Cushing syndrome, rheumatoid arthritis, renal failure, multiple myeloma, and bone metastasis of malignant tumors, were excluded. Commercially available antiosteoporotic drugs in Japan include estrogen, bisphosphonates such as etidronate, alendronate, and risedronate, raloxifene, intramuscular calcitonin, vitamin D_3_, and vitamin K_2_. Studies have implicated several possible metabolic linkages between osteoporosis and vascular calcification involving estrogen deficiency, vitamin D excess, vitamin K deficiency, and lipid oxidation products [5, 15–19]. A recent study also showed that etidronate reduced aortic calcification in patients with end-stage renal disease undergoing chronic haemodialysis [20]. Thus, patients who were being treated with estrogen, bisphosphonates, raloxifene, vitamin D_3_, or vitamin K_2_ were excluded from this study. Plain radiographs of anterior-posterior and lateral views of the thoracic and lumbar spine taken to evaluate posture were available for all the subjects.

The subjects were questioned as to their history of low-traumatic hip fractures and their responses were confirmed using plain radiographs of the hip joint. The prevalence of vertebral fractures was assessed using radiographs of the lateral view of the thoracic and lumbar spine according to the Japanese criteria. The spondylosis grade was also assessed according to the Nathan degree using radiographs of the anterior-posterior view of the lumbar and thoracic spine. The severity of abdominal aortic calcification was assessed using radiographs of the lateral view of the lumbar spine. The associations of sex, age, history of hip fracture, prevalence of vertebral fracture, and spondylosis grade with the severity of aortic calcification were analyzed. Informed consent was obtained from each participant prior to his/her participation in the exercise program. The study protocol was approved by the Ethics Committee of Keiyu Orthopaedic Hospital.

### 2.2. Assessment of Prevalent Vertebral Fracture

**Figure 1:**
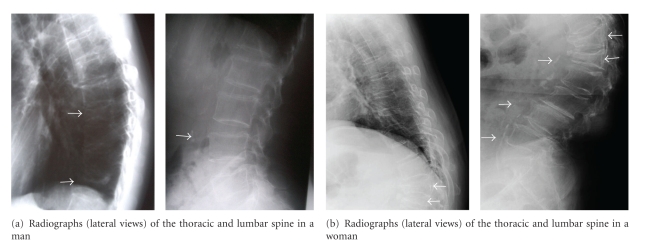
Radiographs (lateral views) of the thoracic and lumbar spine in a man (a) and a woman (b). Abdominal aortic calcification is prominently displayed on routine lateral lumbar spine radiographs as the dense calcium mineral deposits in the aorta of subjects with osteoporotic vertebral fractures.

Plain lateral X-ray films of the thoracic and lumbar spine were used to detect evidence of vertebral fracture (Figure 1). According to the Japanese criteria, vertebral fracture is defined according to the vertebral height on lateral X-ray films [21, 22]. Briefly, vertebral height is measured at the anterior (A), central (C), and posterior (P) parts of the vertebral body, and the presence of a vertebral fracture is confirmed when (1) a reduction in the vertebral height of more than 20% (A, C, and P) as compared with the height of the adjacent vertebrae is observed, (2) the C/A or C/P is less than 0.8, or (3) the A/P is less than 0.75. The assessment of vertebral fractures was performed at the T4-L4 level.

### 2.3. Spondylosis Grade

Plain anterior-posterior X-ray films of the thoracic and lumbar spine were used to detect evidence of spondylosis. The Nathan degree was used to assess the spondylosis grade [23]. First-degree osteophytes appear only as isolated points of initial hyperostosis (grade 1 spondylosis), while second-degree osteophytes are bone protrusions projecting more or less horizontally from the vertebral body (grade 2 spondylosis). In the third-degree category, the osteophytes assume a characteristic “bird's beak” shape that is frequently visible on roentogenograms, with the free end of the “beak” curving in the direction of the intervertebral disc and often coming more-or-less into close contact with the free ends of the osteophytes on the adjacent vertebra (grade 3 spondylosis). The fourth degree is considered to be the stage at which the osteophytes of two adjacent vertebrae become fused together, thereby forming a bone bridge across the intervening intervertebral disc and immobilizing the corresponding intervertebral joint (grade 4 spondylosis). In the present study, subjects with no osteophytes in any vertebra of the thoracic and lumbar spine were judged as having grade 0 spondylosis.

### 2.4. Severity of Abdominal Aortic Calcification

Plain lateral X-ray films of the lumbar spine were used to detect evidence of abdominal aortic calcification (Figure 1). The severity of abdominal aortic calcification was evaluated by measuring the length of the dense calcium mineral deposits in the aorta, which was determined as the number of vertebral bodies along which the calcification ranged.

### 2.5. Statistical Analysis

Data were expressed as the mean  ± standard deviation (SD) in Tables 1 and 2 and in Figure 3. Data comparisons among groups were performed using an analysis of variance (ANOVA) with Fisher's protected least significant difference (PLSD) test. The ratio of men to women and the number of subjects with prevalent vertebral fractures and a history of hip fracture were compared between the two groups using the Fisher exact test. All statistical analyses were performed using the Stat View-J5.0 program on a Windows computer. A significance level of *P* < .05 was used for all comparisons.

## 3. Results

### 3.1. Characteristics of the Study Subjects

Table 1 shows the characteristics of the study subjects. The mean age of the subjects was 73.8 years (range: 48–92 years). Nine subjects (2.2%) had a history of hip fracture, and 221 (55.1%) had at least one prevalent vertebral fracture. Two hundred and sixty-seven subjects (66.6%) had first-degree spondylosis. The mean number of prevalent vertebral fractures was 1.50 (range: 0–13), the mean spondylosis grade was 1.36 (range: 0–4), and the mean severity of abdominal aortic calcification was 1.35 (range: 0–7).

Table 2 shows the characteristics of the study subjects according to the presence of abdominal aortic calcification. The subjects with abdominal aortic calcification were significantly older than the subjects without abdominal aortic calcification (mean age: 75.9 years versus 72.2 years, *P* < .0001). Of the 175 subjects with abdominal aortic calcification, 7 (4.0%) had a history of hip fracture and 129 (73.7%) had at least one prevalent vertebral fracture. Of the 226 subjects without abdominal aortic calcification, 2 (0.9%) and 92 (40.7%) had a history of hip fracture and at least one prevalent vertebral fracture, respectively. The number of subjects with a history of hip fracture and the number of subjects with at least one prevalent vertebral fracture varied significantly between subjects with abdominal aortic calcification and those without abdominal aortic calcification (*P* < .05 and *P* < .0001, resp.). The number of prevalent vertebral fractures was significantly higher among subjects with abdominal aortic calcification than among subjects without abdominal aortic calcification (mean number: 2.20 versus 0.97, *P* < .0001). However, the spondylosis grade did not differ significantly between subjects with abdominal aortic calcification and those without abdominal aortic calcification (mean grade: 1.40 versus 1.34). The mean severity of abdominal aortic calcification was 3.10 among the subjects with abdominal aortic calcification.

### 3.2. Distribution of Vertebral Fracture

**Figure 2:**
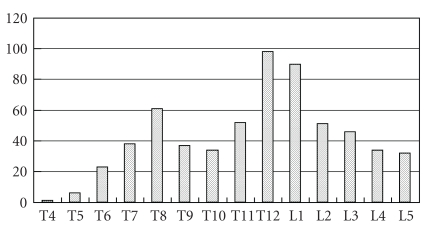
Distribution of vertebral fractures. Two peaks in the distribution of vertebral fractures were observed: the number of vertebral fractures was greatest in the 12th thoracic and 1st lumbar spine, followed by the 8th thoracic spine.

Figure 2 shows the distribution of vertebral fractures. Two peaks in the distribution of vertebral fractures were observed: the number of vertebral fractures was the greatest in the 12th thoracic and 1st lumbar spine, followed by the 8th thoracic spine.

### 3.3. Factors Affecting the Severity of Abdominal Aortic Calcification

**Figure 3:**
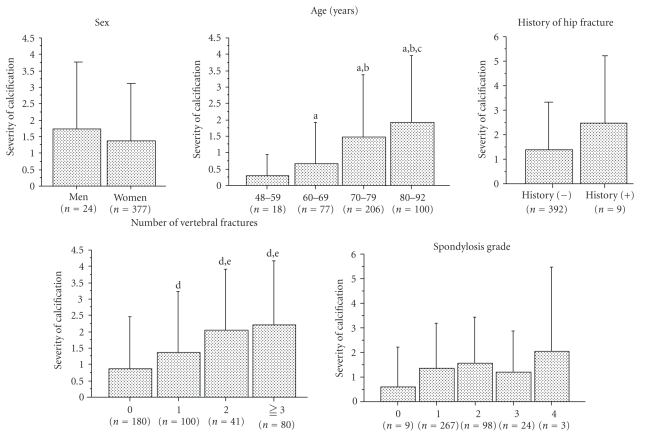
Relations between the severity of abdominal aortic calcification and five factors. Data are expressed as the mean ± standard deviation (SD). Data comparisons among groups were performed using an analysis of variance (ANOVA) with Fisher's protected least significant difference (PLSD) test. (a) Significant versus the 48–59 years of age group, (b) significant versus the 60–69 years of age group, (c) significant versus the 70–79 years of age group, (d) significant versus the no prevalent vertebral fracture group, and (e) significant versus the one prevalent vertebral fracture group.

Figure 3 shows relations between the severity of abdominal aortic calcification and five factors including sex, age, history of hip fracture, number of vertebral fracture, and spondylosis grade. An age-related increase in the severity of abdominal aortic calcifications was observed. The severity of abdominal aortic calcifications was greater among subjects with ≥1 prevalent vertebral fractures than among subjects without any prevalent vertebral fracture. The severity of abdominal aortic calcification was also greater in subjects with ≤2 prevalent vertebral fractures than in subjects with 1 prevalent vertebral fracture. Among the spondylosis grades, however, the severity of abdominal aortic calcification did not differ between men and women or between subjects with and those without a history of hip fracture.

## 4. Discussion

Several hypotheses have been proposed by Farhat et al. [6] to explain the association between aortic calcification, a surrogate marker of atherosclerosis, in terms of cardiovascular disease and osteoporosis including (1) the age-related independent progressions of these diseases, (2) the presence of shared risk factors (such as smoking and physical inactivity), (3) the presence of common pathological mechanisms that could lead to the development of both conditions and that may involve endogenous hormones or inflammatory cytokines, and (4) a cause-effect relationship whereby one condition may lead to the other. However, these hypotheses remain unconfirmed because numerous controversial reports exist regarding this issue [4–10, 12, 24, 25]. The present cross-sectional cohort study confirmed that age and the number of vertebral fractures, but not sex, history of hip fracture, or spondylosis grade, were associated with the severity of abdominal aortic calcification in Japanese postmenopausal women and men.

Although the association of BMD with aortic calcification has been well examined [4–10], the association of non- and low-traumatic fractures with aortic calcification has less thoroughly studied. A few studies have reported a relation between osteoporotic fractures and aortic calcification in healthy postmenopausal European women. Schulz et al. [12] showed that aortic calcification was a strong predictor of vertebral and hip fractures; compared with women without calcification, the Odds ratios (ORs) for vertebral and hip fractures in those with calcification were estimated to be 4.8 and 2.9, respectively. These results regarding vertebral fractures support the results of the present study in Japanese postmenopausal women and men. However, Bagger et al. [4] showed that age (OR: 1.1), body mass index (OR: 0.9), and the severity of aortic calcification (OR: 2.3) were independent predictors of hip fractures, but not of vertebral fractures. These contradictory results regarding vertebral fractures in these two studies might be attributable to the methods used to assess osteoporotic fractures; the former study used a cross-sectional analysis, whereas the latter study used a prospective analysis. Because of the low incidence of hip fractures among Japanese men and women, the present study failed to show an association between abdominal aortic calcification and a history of hip fracture in the Japanese subjects.

In the present study, the severity of abdominal aortic calcification was evaluated by measuring the length of the dense calcium mineral deposits in the aorta, which was determined as the number of vertebral bodies along which the calcification ranged. Previously, Kauppila et al. [26] developed reliable and reproducible indices to classify location, severity, and progression of anterior and posterior calcification in the abdominal aorta. Their summary scores increased with age for both sexes [26]. Although we neither assessed calcification at the anterior and posterior walls of the aorta, nor graded the lesions, the association of age with abdominal calcification was consistent with their results, suggesting that both men and women followed the common progress of aortic atherosclerosis. However, it would be of interest to assess the reliability and reproducibility of the indices developed by Kauppila et al. [26] in Japanese subjects by further work.

Although carotid plaque severity and coronary calcifications were reportedly associated with hand osteoarthritis in women [27], there appear to be very few reports on the association of spondylosis and abdominal aortic calcification in literature. Lumbar spondylosis is degenerative conditions affecting the disks, vertebral bodies, and associated joints of the lumbar spine, which is caused by a progressive and dynamic mechanism [28]. These degenerative changes may appear even in young individuals [28], suggesting that spondylosis is not always associated with aging. Thus, it is unlikely that spondylosis grade related to a progressive and dynamic mechanism may be associated with aortic calcification.

Bakhireva et al. [29] showed the lack of an association between spine or hip BMD and coronary artery calcification in men and women not using hormone therapy and an inverse association between hip BMD and coronary artery calcification in women receiving hormone therapy, suggesting that a possible association between coronary and bone calcium might be mediated by estrogen. However, bone loss and aortic and coronary calcifications are commonly regarded to be more likely to appear during menopause, a time when estrogen production diminishes [3]. Farhat et al. [6] showed that volumetric BMD was associated with high aortic calcification in unadjusted, age-adjusted, and risk-factor adjusted analyses performed using data from healthy middle-aged women, and these results were not influenced by estradiol. In the present study examining 377 postmenopausal women who were not receiving hormone therapy, 164 (43.5%) had abdominal aortic calcifications. Thus, aortic calcifications can be detected even among postmenopausal women who are not receiving.

The most notable limitation of the present study is that several risk factors that were not assessed may be associated with aortic calcification and/or BMD, that is, age, years since menopause, body mass index, level of education, current and previous smoking history, physical activity level, diastolic blood pressure, and low-density lipoprotein cholesterol and triglyceride levels [6, 8]. Most of our subjects were nonsmokers and were sufficiently healthy to participate in the exercise program. Although hypertension and hyperlipidemia are the most important factor for aortic calcification [30, 31], we did not assess these diseases in detail. Thus, further studies are needed to provide further insights into the possible relation between abdominal aortic calcification and the history of hip fracture and the prevalence of vertebral fractures.

In conclusion, the present cross-sectional cohort study showed that age and the number of vertebral fractures, but not sex, history of hip fracture, or spondylosis grade, were significantly associated with the severity of abdominal aortic calcification in Japanese postmenopausal women and men.

## Figures and Tables

**Table 1:** Characteristics of the study subjects.

	Characteristics	Range
Men/Women	24/377	
Age (years)	73.8 ± 7.5	48–92
Number of subjects with a history of hip Fx	9	
Number of subjects with ≥1 prevalent vertebral Fxs	221	
Number of prevalent vertebral Fxs	1.50 ± 2.25	0–13
Spondylosis grade	1.36 ± 0.67	0–4
Severity of calcification (Indicated by the number of the vertebral bodies)	1.35 ± 1.87	0–7

Data are expressed as the mean ± standard deviation (SD). Fx: fractnre.

**Table 2:** Characteristics of the study subjects according to the presence of abdominal aortic calcification.

	Aortic calcification		*P*-value
	Present	Absent	
*N*	175	226	
Men/Women	11/164	13/213	NS
Age (years)	75.9 ± 6.1	72.2 ± 8.0	<.0001
Number of subjects with a history of hip Fx	7	2	<.05
Number of subjects with ≥1 prevalent vertebral Fxs	129	92	<.0001
Number of prevalent vertebral Fxs	2.20 ± 2.47	0.97 ± 1.90	<.0001
Spondylosis grade	1.40 ± 0.63	1.34 ± 0.70	NS
Severity of calcification (Indicated by the number of the vertebral bodies)	3.10 ± 1.62	0.00 ± 0.00	<.0001

Data are expressed as the mean ± standard deviation (SD). Data comparisons between two groups were performed using an analysis of variance (ANOVA) with Fisher's protected least significant difference (PLSD) test. Fx: fracture; NS: not significant.
