# Expression of Concern on “A Radiographic Study on the Associations of Age and Prevalence of Vertebral Fractures with Abdominal Aortic Calcification in Japanese Postmenopausal Women and Men”

**DOI:** 10.1155/2022/9798519

**Published:** 2022-08-02

**Authors:** Journal of Osteoporosis

*Journal of Osteoporosis* would like to express concern with the article titled “A Radiographic Study on the Associations of Age and Prevalence of Vertebral Fractures with Abdominal Aortic Calcification in Japanese Postmenopausal Women and Men” [1]. Potential concerns about this article were raised to our attention due to wider concerns about the integrity of the work of this group [2–4]. Following a reassessment of the article, we asked the authors to respond to the below concerns and provide the relevant documentation where possible:

1. Confirmation that the work was conducted at Keiyu Orthopaedic Hospital, as described in the article.
2. The anonymised patient-level data underlying the results published in the article.
3. A copy of the ethics approval from Keiyu Orthopaedic Hospital.
4. Confirmation of whether any the authors had any financial association with any companies with an interest in fracture treatment or prevention at the time of the study or its publication.

The authors did not respond to the above request. We also asked Keio University to investigate but they were unable to do so because the authors are no longer affiliated to the institution. They stated that Dr. Iwamoto often included his supervisors as co-authors on his papers without their approval, and that Dr. Takeda and Dr. Matsumoto were supervisors of Dr. Iwamoto.
